# Knowledge and attitude of schoolgirls about illegal abortions in Goma, Democratic Republic of Congo

**DOI:** 10.4102/phcfm.v2i1.78

**Published:** 2010-03-11

**Authors:** Lussy J. Paluku, Langalibalele H. Mabuza, Patrick M.H. Maduna, John V. Ndimande

**Affiliations:** 1 Heal Africa Tertiary Hospital of North-Kivu, Goma, Democratic Republic of Congo; 2 Department of Family Medicine and Primary Health Care, University of Limpopo, Medunsa campus, South Africa; 3 Gauteng Department of Health, Head Office, Johannesburg, South Africa

**Keywords:** high school girls, illegal abortions, knowledge, contraceptives, attitude

## Abstract

**Background:**

Adolescent sexual activity, early pregnancy, induced abortion and the increase in HIV infection have become major concerns in sub-Saharan Africa and understanding adolescent sexual behaviour remains a challenge. In the Democratic Republic of Congo (DRC), the practice of illegal abortions is prevalent among school-going adolescent girls with unplanned pregnancies. Assessing their attitude and knowledge on the subject could be a starting point from which to address the problem.

**Objectives:**

To determine the knowledge of schoolgirls in Goma, DRC about the health consequences of illegal abortions and to assess their attitude towards these abortions.

**Method:**

A descriptive cross-sectional study was conducted among a randomly selected sample of 328 high school girls aged 16 to 20 years. A pre-tested, self-administered questionnaire was used for data collection. Nine out of 55 (11 public and 44 private) secondary schools were randomly selected for inclusion in the study. The Epi-Info 2000 computer program was used for data capturing and analysis.

**Results:**

The different sources of information were the radio (66.2%, 217), friends (31.7%, 104), parents (1.5%, 5), and the church (0.5%, 2). The health consequences of illegal abortion mentioned were death, infertility, infection and bleeding. Of the participants, 9.8% (32) had committed an abortion before and 46% (151) knew where to obtain it; 76.2% (250) of participants were against illegal abortion, while 23.8% (78) supported it.

**Conclusion:**

Girls in secondary school in Goma had good knowledge of the illegal abortion practice and its consequences. A fifth of them were in support of the procedure. The DRC government may need to consider legalising abortion to secure a healthy future for affected girls.

## INTRODUCTION

It is estimated that 46 million abortions are performed each year, 20 million of which occur in countries where abortion is prohibited by law.^1^ Adolescent sexual activity and pregnancy are alarmingly common in many countries.^2^ Premature sexual intercourse results in high incidence and prevalence of adolescent pregnancy and abortion and also increases the risk of sexually transmitted infections and, as such, adolescent pregnancy needs careful and proper monitoring to ensure a safe outcome.^3^

Despite the social and cultural importance of child bearing in African society, unwanted pregnancies are a source of problems within the family. This is more acute for adolescent girls who often fall pregnant out of wedlock. Resorting to abortion is commonly their only choice if they wish to avoid facing judgment from their family and community. In Bendel State, Nigeria, attitudes concerning the desirability of abortion were assessed in a survey of 1 805 male and female secondary students. The study showed that, although abortion is still illegal in Nigeria, illegal abortions involving adolescents are widespread, with Catholic students expressing a greater opposition to abortion than protestant students, or those from other religious backgrounds.^4^

Abortion is still illegal in the Democratic Republic of Congo (DRC), except when the life of the mother is in danger. The DRC is a country where the majority of the population practices Catholicism, a religion that discourages all forms of contraception and condemns the termination of pregnancies. Mortality and morbidity associated with illegal abortion have been found to be significant in urban areas such as Goma.^5^

Adolescents represent a significant proportion of the women who choose abortion. The World Health Organisation estimates that at least 33% of all women seeking hospital care for complications related to abortions are under 20 years of age.^6, 7^ Because illegal abortions have high mortality and morbidity rates, legalising abortion is a highly debatable issue among health policy makers worldwide. This study sought to assess the level of knowledge and attitude towards illegal abortion amongst secondary school girls in Goma, DRC.

## METHOD

A descriptive study, using a pre-tested, self-administered questionnaire that had been developed by the research team, was chosen to assess the knowledge and attitude of schoolgirls in Goma about illegal abortion and its consequences. The study population comprised high school girls in Goma aged 16–20. The study population was chosen because it was found to be a high-risk group for unwanted pregnancies and requests for termination of pregnancy.^5, 8^ Both the public and the private school sectors were recruited. Participants were randomly selected; a list of all high schools in Goma was obtained from the provincial inspector of primary and secondary education. There were 55 (11 private and 44 public) secondary schools with fifth and sixth forms in Goma. The town of Goma is divided by three main roads into three sections, with an even distribution of the schools. Although the schools were categorised into the private or public sectors, all the pupils lived in the same town, with almost the same social and cultural norms. Additionally, pupils in private and public schools study the same curriculum, as required by law.

A random sample was obtained, comprising 2 250 girls, aged between 16 and 20, across nine schools (seven private and two public). A representative sample of 328 pupils was derived using a computer statistics software package.^9^ In each of the selected schools, the girls in fifth and sixth form were assembled in a large classroom and provided with a participant information sheet that explained the purpose of this study. Each consenting participant was then requested to sign the consent form. Questionnaires were administered either immediately after school, or during break-time and each participant was required to complete the form in their classroom. Confidentiality was ensured by using an anonymous questionnaire and requesting each participant to sit alone, to avoid influencing others. Completed forms were collected by the principal investigator and the research assistant. The data were captured and analysed using the Epi Info 2000 statistical programme. The study was preceded by a pilot study comprising of 72 respondents from three schools – one public and two private. These respondents were subsequently excluded from the main study. The pilot study assisted in refining the questionnaire.

Approval to conduct the study was granted by the Research, Ethics and Publications Committee (REPC) of the Medical University of Southern Africa (MEDUNSA), now known as the University of Limpopo (Medunsa campus) in South Africa – Clearance Certificate Number: MP 14/2003.

## RESULTS

### Knowledge and sources of knowledge about the health consequences of illegal abortions

In order to assess the participants’ knowledge of illegal abortion, we focussed on the concept of abortion itself, personal experiences (or those of others), the legal implications for abortion in the country, and its influences on an individual's health.

The results regarding the meaning of abortion saw that 61.3% (201) participants knew what abortion meant, 22.6% (74) did not, and 16.1% (53) were unsure. In relation to personal experience, 9.8% (32) of the participants had had an abortion before, 96.9% (31) of whom had induced it, while only one had had a spontaneous abortion; 77.1% (253) of the participants knew someone who had committed an illegal abortion, 22.6% (74) did not, while 0.3% (1) was unsure. The different sources from which they obtained information about abortion-related issues, were the radio (66.2%, 217), friends or colleagues (1.7%, 104), parents (1.5%, 5), and the church (0.5%, 2). Additionally, 46% (151) of participants knew how and where to obtain an abortion. Table 1 demonstrates the knowledge categories and the corresponding frequencies.

**TABLE 1:** Knowledge about illegal abortion (n = 328)

Knowledge category	Item	Number	%
Source of information	Radio	217	66.2
	Friends	104	31.7
	Parents	5	1.5
	Church	2	0.5
Meaning of abortion	Known	201	61.3
	Unknown	74	22.6
	Unsure	53	16.1
Knowledge that it is illegal	Yes	272	82.9
	No	41	12.5
	Unsure	15	4.6
Knew someone who did it	Yes	253	77.1
	No	74	22.6
	Unsure	1	0.3
Personal experience	Total number	32	9.8
	Self-induced	31	9.4
	Spontaneous	1	0.3
Knew where to obtain it	Yes	151	46
	No	159	48.5
	Unsure	18	5.5

Table 2 shows that the majority of the participants were 18 years old 32.6% (107/328), followed by the 19-year-olds 30.8% (101/328), the 17-year-olds 23.8% (78/328), the 16-year-olds 9.8% (32/328), with the lowest category being the 20-year-olds 3% (10/328).

**TABLE 2:** Age and knowledge about illegal abortion (n = 328)

		Age of participants (years)										

		16		17		18		19		20		Total	
					
		Freq	%	Freq	%	Freq	%	Freq	%	Freq	%	Freq	%
Knowledge that abortion is illegal (n = 328)	Yes	9	2.7	63	19.2	94	28.7	97	29.6	9	2.7	**272**	**82.9**
	No	19	5.8	13	4.0	6	1.8	2	0.6	1	0.3	**41**	**12.5**
	Uncertain	4	1.3	2	0.6	7	2.1	2	0.6	0	0.0	**15**	**4.6**

**Total**		**32**	**9.8**	**78**	**23.8**	**107**	**32.6**	**101**	**30.8**	**10**	**3.0**	**328**	**100**

The knowledge level among the girls increased according to their age across their respective age categories: 28.1% (9/32) among the 16-year-olds, 80.8% (63/78) among the 17-year-olds, 87.9% (94/107) among the 18-year-olds, 96.0% (97/101) among the 19-year-olds, and 90.0% (9/10) among the 20-year-olds. Overall, the respective percentages of the girls who had knowledge about illegal abortion according to age groups were 2.7%, 19.2%, 28.7%, 29.6% and 2.7%. The total percentage in this category was 82.9%.

Of the 328 participants, 249 (75.9%) knew someone who became ill after having an abortion. Most participants (83.2%) knew some health consequences of illegal abortion, but 53 participants (16.1%) did not have knowledge of any health consequence of an illegal abortion and two participants (0.7%) were not sure of their knowledge.

**FIGURE 1:**
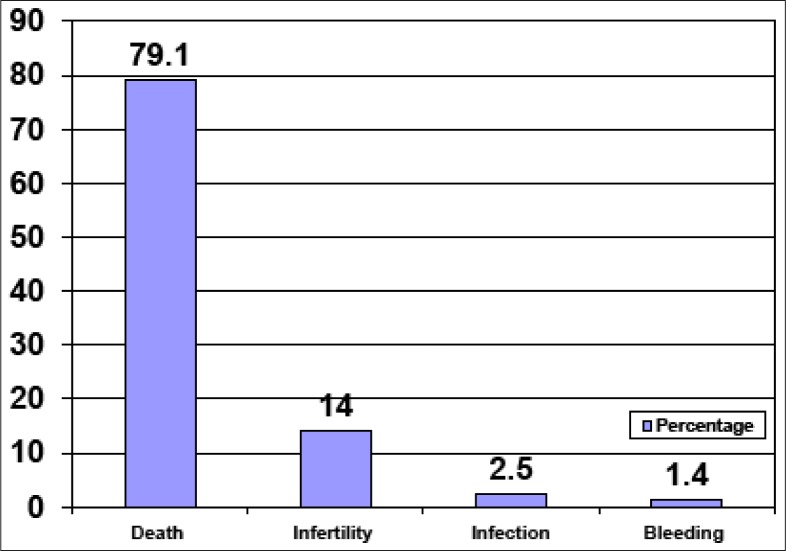
Health consequences of illegal abortion

Figure 1 shows the knowledge of the schoolgirls about the health consequences of illegal abortion. Health consequences mentioned were death (79.1%, 233), infertility (14%, 46), infection (2.5%, 8) and bleeding (1.4%, 5).

### Attitude towards illegal abortion

While most of participants, 76.2% (250), were against illegal abortion, 23.8% (78) supported it. Table 3 shows the reasons the participants gave in support or rejection of illegal abortion. The two main reasons for the participants who supported abortion were unplanned pregnancy 9.5% (7), and to prevent school disruption because the girl was still at school, 8.5% (6). The largest age-group to support illegal abortion was the 18-year-olds, 12.2% (10). For those who did not support illegal abortion, spiritual convictions 34.1% (85) and health problems 34.9% (87) were the main reasons identified. Illegal abortion was almost equally rejected among the 17-, 18- and 19-year-old participants 21.8% (55).

**TABLE 3:** Reasons for or against illegal abortion (n = 328)

	Age (years)	16	17	18	19	20	Total
Reason in support of illegal abortion (n = 78)	Unplanned pregnancy (%)	0.0	1.8	4.1	2.6	1.0	9.5
	Still at school (%)	0.0	1.3	6.1	0.5	0.6	8.5
	Avoid disappointing parents (%)	0.0	0.5	1.0	0.2	0.2	1.9
	Poverty (%)	0.1	0.0	1.0	2.8	0.0	3.9
	**Sub-total (%)**	**0.1**	**3.6**	**12.2**	**6.1**	**1.8**	**23.8**

Reasons against illegal abortion (n = 250)	Spiritual convictions (%)	4.5	13.8	7.1	8.7	0.0	34.1
	Health reasons (%)	5.2	6.0	12.7	9.8	1.2	34.9
	Personal preferences (%)	0.0	0.4	0.6	6.2	0.0	7.2
	**Sub-total (%)**	**9.7**	**20.2**	**20.4**	**24.7**	**1.2**	**76.2**

**Total (%)**							**100**

**FIGURE 2:**
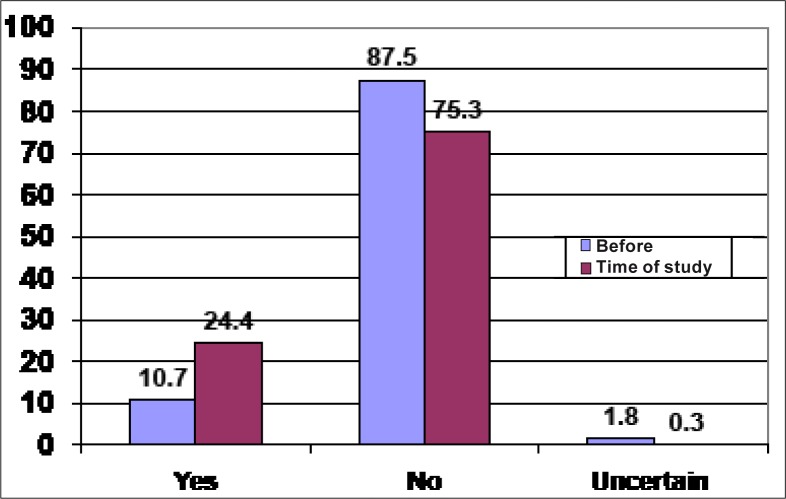
Abortion consideration before and at time of study

Figure 2 shows that 10.7% (35) of the participants had considered illegal abortion in the past and 9.7% (32) (data not shown) of these had committed an illegal abortion. This means that approximately one in ten respondents had considered illegal abortion in the past. This figure also shows that more than double 24.4% (80) of participants who had considered abortion before, would consider it if they were pregnant at the time of the study. This means that approximately two in ten respondents would consider illegal abortion if found pregnant at the time of the study.

## DISCUSSION

### Knowledge of abortion

Across the world, abortion is very often morally defined. The percentage of participants who knew the meaning of abortion indicated that the topic was well known in Goma too. In a study conducted in Korea, it was found that the mass media had informed the public about several sexual problems facing that country, including early sexual intercourse among the youth, unwanted pregnancies and increased rate of induced abortions.^10^ According to the responses obtained with regard to the sources of information on illegal abortion, the Goma community uses mostly the radio to access information. This was supported by a study done in Kenya on the knowledge and perceptions of abortion among adolescents.^11^

Our study demonstrated that parents (1.5%) and the church (0.5%) provide little knowledge to girls on illegal abortion. Parents were also found to play a minor role in delivering information on abortion to adolescents in the study done in Kenya.^12^ An explanation for the limited church involvement in Goma might be that Catholicism is the dominant religion in the country and is known to be conservative regarding sexual matters. On the contrary, the Catholic religion was found to be encouraging education of adolescents about abortion in the Philippines.^12^ Regarding information from the household, it was found that teenagers received very little sex education from their parents in Zambia,^13^ which could be due to the fact that talking about sex is often still regarded as taboo in many African societies, including the DRC.

### Experiential knowledge of abortion

Regarding the experience of schoolgirls on abortion, the study found that 9.7% of the participants reported having had an abortion before, among which 96.9% of them had induced the abortion. This proportion seems to be higher than the observation made in Nairobi, Kenya, where only 1.7% of the 1 751 adolescent schoolgirls – to whom a questionnaire had been administered about their knowledge on reproductive biology, sexual behaviour and its relationship to contraceptive practice – admitted to induced abortion.^14^ On the other hand, findings in this study were lower than those found in a study conducted in Benin City, Nigeria, in which 160 respondents (30.2%) were reported to have committed an illegal abortion.^15^

Because abortion was still illegal in the DRC at the time of the study (2003), many cases of abortion were believed not to have been reported. It could therefore be inferred that the recorded figures were an underestimation. The under-reporting of cases was in keeping with a study on sexual experience, knowledge, attitude and practice of contraception, conducted among senior high school students in north Gonder, Ethiopia. In that study, 30.1% of sexually active female students were found pregnant, among whom only 4.8% admitted to have committed an illegal abortion, whereas the actual figure was most probably higher.^16^

**Figure:**
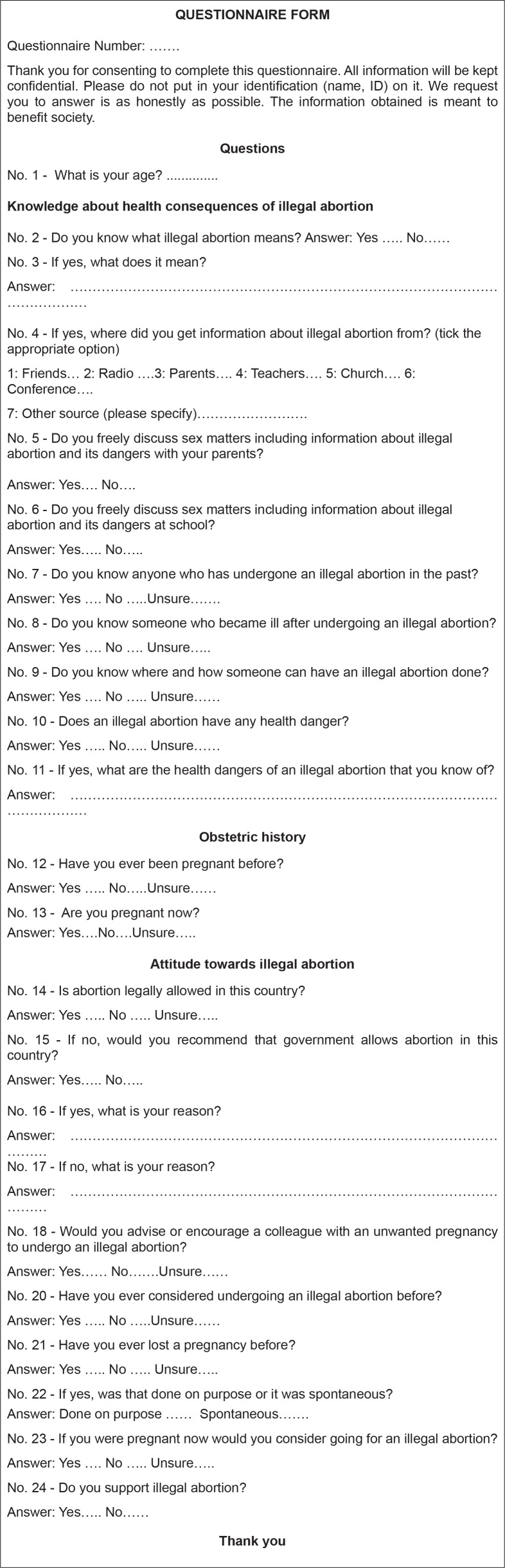


### Knowledge about health consequences of illegal abortion

The study showed that three in five schoolgirls had knowledge about illegal abortion, close to four in five knew someone who had committed an illegal abortion and also that four in five were aware that it was illegal in the DRC. Therefore, the subject of illegal abortion was not new among the participants. As abortion is a criminal offense in the DRC, affected girls tend to procure it illegally, resulting in adverse consequences, as stated by those who had known someone who had committed it before, including themselves.

Regarding the health consequences of illegal abortion, it was found that death was the most mentioned consequence, followed by infertility, infection and bleeding. In his article entitled *Youth often risk unsafe abortions*, Barnett identified infection, mortality, haemorrhage and infertility as serious health problems associated with unsafe abortion. He showed that because adolescents lacked the knowledge of where to go for a safe procedure, they were more likely to become victims of these complications. Furthermore, in the same study, he found that the risk of complications was higher for adolescents.6 Similar complications were reported in another study on adolescents and abortion done in Dar-es-salam, Tanzania.^17^

It would be difficult to estimate the number of girls who die as a result of illegal abortions in the DRC because no study has ever been conducted in this regard. Nevertheless, the WHO currently estimates that there are 115 068 unsafe abortions undertaken daily across the world. About 95% of these occur in the developing countries, such as the DRC, and lead to the deaths of more than 200 women daily.^5, 18^ Complications of abortion include infertility due to tubal damage, pelvic infection and chronic pelvic disease.^19^

### Attitude towards abortion and its health consequences

Most participants (76.2%) were against illegal abortion, while 23.8% supported it. The girls knew that it could lead to death, infertility, infection and bleeding. Reasons given against illegal abortion were spiritual convictions, health reasons and personal preferences. On the legalisation of abortion, our findings were almost similar to those found in Benin City, where a significant number of the schoolgirls had resorted to illegal abortion as a solution to their unwanted pregnancy. Nevertheless, in that study the percentage was slightly higher (30%) than the one in our study.^15^ In our study, reasons given in support of the illegal abortion were the fact that the pregnancy was unplanned, the girl was still at school, to avoid disappointing parents, and lack of proper resources to take care of the girl and the baby during the pregnancy and after. In the DRC, abortion is only allowed on medical grounds, that is, to save the life of the mother. Similar reasons for procuring an illegal abortion, such as to avoid being expelled or forced to drop out of school, were given in the Nigerian study mentioned above.^15^ In our study, approximately one in ten of the respondents had considered illegal abortion in the past.

This study showed that more than double (24.4% vs 10.7%) of participants (80 vs 35 participants) who have considered abortion before would again consider it if they were pregnant at the time of the study. These findings suggest that the number of schoolgirls who would consider abortion is on the rise. Previous studies have already demonstrated that abortion is becoming endemic, particularly among adolescents who are attending secondary school.^20^ The WHO confirmed this by showing that sexual activity is increasing among adolescents and by demonstrating that, in many African countries, up to 70% of all women hospitalised for abortion complications are younger than 20 years old.^5^ As long as adolescents are involved in sexual activities, they are at a high risk of becoming pregnant. Studies in Africa have found that accessibility to family planning information and services is lacking.^6, 15^

## CONCLUSION

The schoolgirls of Goma, DRC had a reasonable knowledge of illegal abortions, their common source being the radio and friends. Almost half of the respondents knew how and where to obtain an abortion; the other half were ignorant in this regard. Most of those who have had an abortion had induced it. The majority of the girls knew that abortion was illegal in the country. They also knew that illegal abortion could lead to death, infertility, infection and bleeding. However, in spite of their knowledge about abortion and health consequences of illegal abortion, the willingness to consider abortion was found to be increasing among schoolgirls in Goma. The Congolese Government may need to consider legalising abortion to secure a healthy future for affected girls.
